# Fluid hydration to prevent post-ERCP pancreatitis in average- to high-risk patients receiving prophylactic rectal NSAIDs (FLUYT trial): study protocol for a randomized controlled trial

**DOI:** 10.1186/s13063-018-2583-x

**Published:** 2018-04-02

**Authors:** Xavier J. N. M. Smeets, David W. da Costa, Paul Fockens, Chris J. J. Mulder, Robin Timmer, Wietske Kievit, Marieke Zegers, Marco J. Bruno, Marc G. H. Besselink, Frank P. Vleggaar, Rene W. M. van der Hulst, Alexander C. Poen, Gerbrand D. N. Heine, Niels G. Venneman, Jeroen J. Kolkman, Lubbertus C. Baak, Tessa E. H. Römkens, Sven M. van Dijk, Nora D. L. Hallensleben, Wim van de Vrie, Tom C. J. Seerden, Adriaan C. I. T. L. Tan, Annet M. C. J. Voorburg, Jan-Werner Poley, Ben J. Witteman, Abha Bhalla, Muhammed Hadithi, Willem J. Thijs, Matthijs P. Schwartz, Jan Maarten Vrolijk, Robert C. Verdonk, Foke van Delft, Yolande Keulemans, Harry van Goor, Joost P. H. Drenth, Erwin J. M. van Geenen, for the Dutch Pancreatitis Study Group

**Affiliations:** 1 Department of Gastroenterology and Hepatology, Radboud University Medical Centre, PO 9101, 6500 HB Nijmegen, The Netherlands; 2 Department of Radiology, St Antonius Hospital, PO 2500, 3430 EM Nieuwegein, The Netherlands; 3 Department of Gastroenterology and Hepatology, Academic Medical Centre, PO 22660, 1100 DD Amsterdam, The Netherlands; 4 Department of Gastroenterology and Hepatology, VU University Medical Centre Amsterdam, PO Box 7057, 1007 MB Amsterdam, The Netherlands; 5 Department of Gastroenterology and Hepatology, St Antonius Hospital, PO 2500, 3430 EM Nieuwegein, The Netherlands; 6 Department of Health Evidence, Radboud University Medical Centre, PO 9101, 6500 HB Nijmegen, The Netherlands; 7 Radboud Institute for Health Sciences, IQ Healthcare, Radboud University Medical Centre, PO 9101, 6500 HB Nijmegen, The Netherlands; 8 Department of Gastroenterology and Hepatology, Erasmus Medical Centre, PO 2040, 3000 CA Rotterdam, The Netherlands; 9 Department of Surgery, Academic Medical Centre, PO 22660, 1100 DD Amsterdam, The Netherlands; 10 Department of Gastroenterology and Hepatology, University Medical Centre Utrecht, PO 85500, 3508 GA Utrecht, The Netherlands; 11 Department of Gastroenterology and Hepatology, Spaarne Gasthuis, PO 417, 2000 AK Haarlem, The Netherlands; 12 Department of Gastroenterology and Hepatology, Isala Klinieken, PO 10400, 8000 GK Zwolle, The Netherlands; 13 Department of Gastroenterology and Hepatology, Noord-West Hospital, PO 501, 1800 AM Alkmaar, The Netherlands; 14 Department of Gastroenterology and Hepatology, Medisch Spectrum Twente, PO 50000, 7500 KA Enschede, The Netherlands; 15 Department of Gastroenterology and Hepatology, Onze Lieve Vrouwe Gasthuis, Postbus 95500, 1090 HM Amsterdam, The Netherlands; 16 Department of Gastroenterology and Hepatology, Jeroen Bosch Hospital, PO 90153, 5200 ME s’Hertogenbosch, The Netherlands; 17 Department of Gastroenterology and Hepatology, Albert Schweitzer Hospital, PO 444, 3300 AK Dordrecht, The Netherlands; 18 Department of Gastroenterology and Hepatology, Amphia Hospital, PO 90158, 4800 RK Breda, The Netherlands; 19 Department of Gastroenterology and Hepatology, Canisius-Wilhelmina Hospital, PO 9015, 6500 GS Nijmegen, The Netherlands; 20 Department of Gastroenterology and Hepatology, Diakonessenhuis, PO 80250, 3508 TG Utrecht, The Netherlands; 21 Department of Gastroenterology and Hepatology, Hospital Gelderse Vallei, PO 9025, 6710 HN Ede, The Netherlands; 22 Department of Gastroenterology and Hepatology, HAGA Hospital, PO 40551, 2504 LN The Hague, The Netherlands; 23 Department of Gastroenterology and Hepatology, Maasstad Hospital, PO 9100, 3007 AC Rotterdam, The Netherlands; 24 Department of Gastroenterology and Hepatology, Martini Hospital, PO 30033, 9700 RM Groningen, The Netherlands; 25 Department of Gastroenterology and Hepatology, Meander Medical Centre, PO 1502, 3800 BM Amersfoort, The Netherlands; 26 Department of Gastroenterology and Hepatology, Rijnstate Hospital, PO 9555, 6800 TA Arnhem, The Netherlands; 27 Department of Gastroenterology and Hepatology, Zuyderland, PO 5500, 6130 MB Sittard-Geleen, The Netherlands; 28 Department of Surgery, Radboud University Medical Centre, PO 9101, 6500 HB Nijmegen, The Netherlands

**Keywords:** Post-ERCP pancreatitis, Prevention, ERCP, Hydration, NSAIDs

## Abstract

**Background:**

Post-endoscopic retrograde cholangiopancreatography (ERCP) pancreatitis (PEP) is the most common complication of ERCP and may run a severe course. Evidence suggests that vigorous periprocedural hydration can prevent PEP, but studies to date have significant methodological drawbacks. Importantly, evidence for its added value in patients already receiving prophylactic rectal non-steroidal anti-inflammatory drugs (NSAIDs) is lacking and the cost-effectiveness of the approach has not been investigated. We hypothesize that combination therapy of rectal NSAIDs and periprocedural hydration would significantly lower the incidence of post-ERCP pancreatitis compared to rectal NSAIDs alone in moderate- to high-risk patients undergoing ERCP.

**Methods:**

The FLUYT trial is a multicenter, parallel group, open label, superiority randomized controlled trial. A total of 826 moderate- to high-risk patients undergoing ERCP that receive prophylactic rectal NSAIDs will be randomized to a control group (no fluids or normal saline with a maximum of 1.5 mL/kg/h and 3 L/24 h) or intervention group (lactated Ringer’s solution with 20 mL/kg over 60 min at start of ERCP, followed by 3 mL/kg/h for 8 h thereafter). The primary endpoint is the incidence of post-ERCP pancreatitis. Secondary endpoints include PEP severity, hydration-related complications, and cost-effectiveness.

**Discussion:**

The FLUYT trial design, including hydration schedule, fluid type, and sample size, maximize its power of identifying a potential difference in post-ERCP pancreatitis incidence in patients receiving prophylactic rectal NSAIDs.

**Trial registration:**

EudraCT: 2015-000829-37. Registered on 18 February 2015.

ISRCTN: 13659155. Registered on 18 May 2015.

**Electronic supplementary material:**

The online version of this article (10.1186/s13063-018-2583-x) contains supplementary material, which is available to authorized users.

## Background

Endoscopic retrograde cholangiopancreatography (ERCP) is widely used to treat diseases of the pancreaticobiliary tree. The most frequent complication is post-ERCP pancreatitis (PEP) [1]. The reported overall incidence varies from 7% to 10% and approaches 15% in high-risk patients [2]. In the United States, costs related to PEP are estimated to be over $200 million annually [3].

Numerous prophylactic measures for PEP have been investigated [4]. However, the evidence is indisputable for only two measures – rectal non-steroidal anti-inflammatory drugs (NSAIDs) and prophylactic pancreatic duct (PD) stents. Recent meta-analyses calculated an odds ratio of 0.44 for rectal NSAIDs [5] and 0.35 for PD stents [6]. Therefore, the American Society for Gastrointestinal Endoscopy recommends the use of rectal NSAIDs and PD stents in high-risk patients and suggests the use of rectal NSAIDs in average-risk patients [7]. The European Society for Gastrointestinal Endoscopy (ESGE) recommends routine use of rectal NSAIDs in all patients undergoing ERCP, while reserving PD stents for high-risk patients [4].

A new promising prophylactic strategy for PEP is periprocedural hydration. It is intended to preserve adequate pancreatic perfusion and tissue oxygenation during ERCP. The strategy finds its justification in the theory that early pancreatic microcirculatory perfusion derangements are correlated with severity of acute pancreatitis [8]. Circumstantial evidence supporting this theory is that an increased level of pre-procedural blood urea nitrogen, a marker of hemoconcentration, has been associated with PEP development and severity [9, 10]. Many patients subjected to ERCP are fasting and may therefore be relatively dehydrated. Furthermore, a retrospective cohort study found an inverse relationship between peri-ERCP hydration and PEP severity [11].

A recent meta-analysis on periprocedural hydration [12], including seven RCTs with 1047 patients, showed an odds ratio of 0.47 (0.30–0.72; *P* = 0.0006) in favor of protection against PEP. There was no significant difference in adverse events between the intervention and control groups (*P* = 0.23). However, the included RCTs had several shortcomings, the most important of which being that patients did not receive rectal NSAIDs – these cannot be withheld from patients due to the clear evidence in favor of their use [13]. Furthermore, a synergistic effect of hydration and rectal NSAIDs is plausible because both act at a different stage of PEP development; hydration preserves pancreatic microcirculation and NSAIDs suppress the inflammatory response.

The FLUYT trial is designed to investigate whether periprocedural hydration with lactated Ringer’s solution can prevent post-ERCP pancreatitis in moderate- to high-risk patients undergoing ERCP who already receive prophylactic rectal NSAIDs.

## Methods

**Fig. 1:**
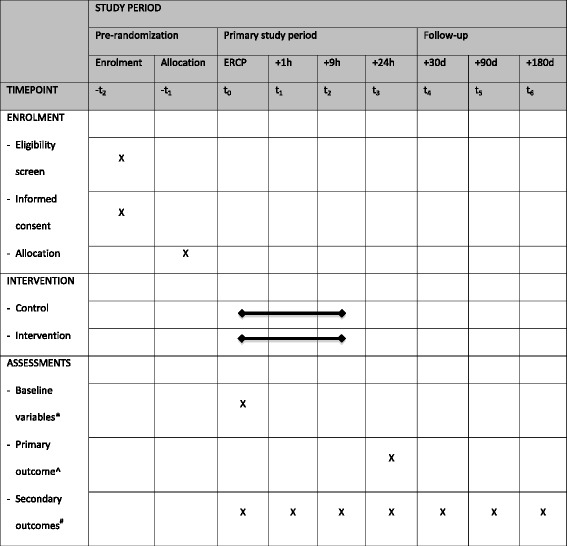
SPIRIT schedule of enrolment, interventions, and assessments. *Baseline variables: age, sex, comorbidity, American Society of Anesthesiologists score, ERCP indication, PEP risk factors, use of pancreatic duct stents. ^Primary outcome: post-ERCP pancreatitis. ^#^Secondary outcomes: incidence of delayed PEP (> 24 h after ERCP), severity of PEP, other ERCP complications, hydration-related complications, length of hospital and intensive care unit stay, health-related quality of life, cost-effectiveness, and exocrine and endocrine pancreatic insufficiency

The trial protocol is written in accordance with the SPIRIT guidelines (Fig. 1, Additional file 1) [14].

### Study setting

The FLUYT trial is a multicenter, parallel group, open label, superiority randomized controlled trial that will include 826 patients from 20 hospitals of the Dutch Pancreatitis Study Group, including 3 university medical centres and 17 large teaching hospitals (see ‘Participating Centers’ at the end of the protocol for more details).

### Eligibility criteria

Patients aged 18–85 years undergoing ERCP and who provide written informed consent are included in the study.

The exclusion criteria are as follows:

- Low risk of post-ERCP pancreatitis: (1) definite chronic pancreatitis according to MANNHEIM criteria [15], (2) previous sphincterotomy, (3) pancreatic head mass, and (4) routine biliary stent exchange. In case of a pancreatic duct intervention, chronic pancreatitis and previous sphincterotomy are not exclusion criteria
- Acute pancreatitis
- Altered anatomy, defined as anatomical variations in which bile and/or pancreatic secretions (in case of pancreatic duct interventions) do not enter the duodenum by way of the ampulla of Vater (e.g., Roux-en-Y reconstruction, surgery for chronic pancreatitis)
- Pregnancy
- Signs of congestive heart failure, such as pitting edema or a New York Heart Association classification greater than class I heart failure
- Respiratory insufficiency (pO_2_ < 60 mmHg or saturation < 90% despite FiO_2_ of 30% or requiring mechanical ventilation)
- Severe liver disease (cirrhosis and ascites)
- Patients receiving more than 1.5 mL/kg/h or 3 L/24 h of intravenous fluids in the 24 h before ERCP
- Hypotension (systolic blood pressure < 90 mmHg or mean arterial pressure < 70 mmHg)
- Hypo- or hypernatremia (serum Na^+^ levels < 130 or > 150 mmol/L)
- Contraindications for rectal NSAIDs, including allergy, active gastrointestinal bleeding, ulcer disease, renal insufficiency (glomerular filtration rate < 30 mL/min) and NSAID use for other indications (other than cardioprotective aspirin)

### Treatment arms and co-interventions

Eligibility of all potential participants will be discussed with the central study coordinator. After written informed consent, patients are randomized to either the control or intervention group. The control group will be administered 100 mg of indomethacin or diclofenac within 30 min before or after ERCP and no hydration or mild hydration with normal saline, with a maximum of 1.5 mL/kg/h or 3 L/24 h;. After 24 h, the volume and nature of intravenous infusion is at the discretion of the treating physician. The intervention group will be administered 100 mg of indomethacin or diclofenac within 30 min before or after ERCP and periprocedural hydration with lactated Ringer’s solution, 20 mL/kg within 60 min from the start of ERCP (endoscope-mouth contact), directly followed by 3 mL/kg/h for 8 h. Thereafter, the volume and nature of intravenous infusion is at the discretion of the treating physician.

The type, dosing, and timing of rectal NSAID application follow the recommendations of the ESGE [4]. Current ERCP guidelines give no indication for hydration in the control group. Therefore, the hydration schedules in both the intervention and control group are based on the favorable results seen in Buxbaum’s pilot study [16].

To ensure timely delivery of the 60 min bolus, pressure bags or double infusion pumps will be used. The hydration is maximized in patients with morbid obesity (body mass index > 40), because their altered physiology is characterized by a decrease of lean body in tissue water content [17]. Therefore, in these patients, a maximum amount of fluid is calculated by using a fictive maximum weight (FMW) associated with a body mass index of 40 kg/m^2^. The FMW is calculated as follows:

$$ FMW=40\ {\left( patient\ length\ in\ meters\right)}^2 $$

The FMW is inserted in the fluid equations of the intervention group.

If the clinical condition of patients in the control group does not allow the infusion restrictions (e.g., in case of hypovolemic shock), higher volume infusion is allowed at the discretion of the treating physician. Furthermore, if patients in the intervention group develop signs of fluid overload, the intensive hydration will be stopped and, if needed, diuretics will be started.

General treatment measures include a fasting state before ERCP. Antibiotic prophylaxis, measures to correct coagulation disorders and diet reintroduction after endoscopic sphincterotomy will be managed according to a local protocol. We did not encourage the use of pancreatic duct stents to prevent confounding with the intensive hydration regimen. After ERCP, all patients will be hospitalized for a minimum stay of 24 h for timely diagnosis of adverse events and monitoring of intravenous fluid volumes. Longer monitoring, hospitalization, and treatment of adverse events is at the discretion of the treating physician. Post-ERCP pancreatitis will be treated in accordance with the International Association of Pancreatology/American Pancreatic Association guidelines for treatment of acute pancreatitis [18].

To improve adherence to the trial protocol, involved staff members (gastroenterologists, residents, physician assistants, endoscopy nurses, ward nurses and sedationists) in all participating centres will receive specific training in the trial’s standard operating procedures (SOPs). In coordination with the local principal investigator, the SOPs are adapted to the local hospital setting, while ensuring adherence to the trial protocol. Furthermore, the SOPs will be readily available on all wards and on the dedicated FLUYT trial website. The trial coordinator is on call 24/7 to assist in case of any questions. Protocol adherence is evaluated regularly in participating centres.

### Outcomes

The primary outcome is the incidence of post-ERCP pancreatitis according to the Cotton criteria [19]. The criteria of (1) new onset upper abdominal pain; (2) elevation of pancreatic enzymes (amylase and/or lipase) to more than three times the institutional upper limit of normal; (3) criteria 1 and 2 are present at least 24 h after ERCP; and (4) hospitalization (or extension of planned admission) for at least 2 nights, must all be present.

The secondary endpoints (Additional file 1) are (1) incidence of delayed PEP (PEP occurring > 24 h after the procedure); (2) severity of PEP – this will be reported according to the Cotton criteria [19] and the revised Atlanta criteria [20] since the severity grading differs between the two classifications [21, 22] and both are reported in studies; (3) ERCP-related complications according to the Cotton criteria [19], namely bleeding, perforation, and infection; (4) hydration-related complications, such as pulmonary edema and congestive heart failure; (5) length of hospital and intensive care unit stay; (6) generic health-related quality of life, measured by the EQ-5D and SF-36 [23, 24]; (7) cost-effectiveness; and (8) exocrine (fecal elastase-1 < 200 μg/L) and endocrine (HbA1c > 42 mmol/L) pancreatic insufficiency.

### Sample size calculation

A recent meta-analysis [25] reported an 8% PEP incidence in patients receiving prophylactic rectal NSAIDs. We believe periprocedural hydration is a useful addition to rectal NSAIDs if it has a similar relative risk reduction of 60% [25, 26]. This minimal clinically important difference will cause the incidence of PEP to decrease from 8% in the control group to 3.2% in the intervention group, with a 4.8% absolute risk reduction. With a two-sided significance level of 5% and a power of 80%, a total of 718 patients (359 per treatment arm) is required to demonstrate this effect. To account for drop-out and missing data, we increased the sample size by 15%. This amounts to a final number of 826 patients (413 per treatment arm).

### Randomization

Patients are randomized centrally by the study coordinator in a 1:1 ratio by using a web-based randomization module. Participants were stratified by center. Within each stratum, random block sizes of 2, 4, and 6 were used. Due to the large sample size, age and sex are expected to be distributed equally between the groups. Therefore, no additional strata are used.

### Blinding

Patients and treating physicians are not blinded for treatment allocation (see Discussion). However, a blinded adjudication committee will assess and weigh all events (severe complications and mortality) and decide whether the pre-specified definitions of the primary and secondary endpoints are met. The adjudication committee consists of six gastroenterologists with extensive ERCP experience, a radiologist and a nephrologist. On the basis of primary source data, each member will individually evaluate a patient’s disease course. Disagreements are resolved in a plenary consensus meeting. A final analysis will only be performed after consensus has been reached on each individual endpoint for each individual patient.

### Data collection methods and follow-up

Clinical data are collected locally on standardized digital case record forms (CRFs) before ERCP, directly afterwards and in the 24 h thereafter. CRFs were created for the endoscopist, nursing staff, and treating physician on which to score the occurrence of the primary and secondary endpoints. Endoscopy and ward nurses will monitor all intravenous fluid infusion during the first 24 h after ERCP. After 24 h, the treating physician will assess the primary endpoint (abdominal pain suggestive of PEP) and blood is drawn for measurement of serum amylase and lipase. To ensure data quality, the central study coordinator will check all CRFs and contact responsible staff members in case of inconsistencies.

All patients are followed up for 180 days after randomization. Patients are contacted by telephone after 30, 90, and 180 days by a trial nurse. The validated EQ-5D, SF-36, and iMTA PCQ questionnaires for measuring quality of life and indirect non-medical costs will be sent simultaneously by (e-)mail, with a telephone reminder after a week if there is no response [23, 24, 27]. After 1 month of non-response, another telephone reminder will follow and a new questionnaire will be sent. In case of hospitalization, patients will be interviewed by a ward nurse. If patients experienced post-ERCP pancreatitis, pancreatic function will be assessed at 180 days post-randomization by fecal elastase and serum HbA1c measurements.

Unblinded, independent monitors will visit participating sites yearly for source document verification of 10% of the CRFs, including all components of the primary endpoint. If inconsistencies are encountered, all CRFs will be inspected.

### Statistical methods

#### Descriptive statistics

The baseline characteristics of age, sex, comorbidity, American Society of Anesthesiologists score, ERCP indication, PEP risk factors according to ESGE [4], and the use of other prophylactics (mainly pancreatic duct stents) will be reported. Data will be presented in percentages for categorical variables. Continuous variables will be presented as mean with standard deviation (normal distribution) or median with interquartile range (skewed distribution).

#### Primary analysis

The primary endpoint will be analyzed according to the intention-to-treat principle with the use of Fisher’s exact test. That is, all randomized patients will be analyzed according to their original treatment allocation, regardless of study protocol violations. The only patients excluded from the analysis will be those in whom the duodenum was not reached and the papilla was not manipulated (e.g., in case of upper gastrointestinal stenosis, aspiration risk, restless patients). Because these patients did not have an ERCP, there is no risk of PEP. Comparison of the primary endpoint will be expressed in terms of a relative risk and 95% confidence intervals. An exploratory per-protocol analysis will also be performed. Reasons for protocol violations will be described. In these analyses we will not adjust for stratification by site. A two-tailed *P* value of less than 0.05 is considered to be statistically significant.

#### Additional analyses

The secondary endpoints will be compared between treatment groups by the Student’s *t* test, Wilcoxon rank sum test, Pearson’s χ^2^ test, or Fischer exact test as appropriate. We cannot rule out the possibility that pancreatic duct stents are placed. If that scenario plays out, we will perform a sensitivity analysis of the primary endpoint in two subgroups, namely patients that only received rectal NSAIDs and patients that received combination therapy with pancreatic duct stents. Furthermore, we will conduct a sensitivity log-binomial regression analysis of our primary endpoint in which we adjust for stratification by site. Finally, the costs and effects of both treatment strategies within the 6 months of follow-up will be compared. Cost-effectiveness will be expressed as costs per patient with poor outcome (severe morbidity and/or death) and costs per quality adjusted life year up to 180 days after randomization. Healthcare costs are registered on structured CRFs. Unit prices according to the handbook of the Dutch Health Council are used [28, 29]. Productivity costs are measured by iMTA PCQ and quality adjusted life years by the EQ-5D questionnaire [24, 27]. The cost-effectiveness analysis will be reported separately from the primary study manuscript.

### Safety

All adverse events, regardless of a supposed connection to the trial, will be reported to the study coordinator. In turn, the coordinator reports adverse events to the Central Committee on Research Involving Human Subjects (CCMO) according to the CCMO directive (death within 24 h, other serious adverse events within 15 days after the sponsor has first knowledge of the event). All serious adverse events will be followed until they have abated or until a stable situation has been reached.

To monitor patient recruitment and safety, an independent data safety monitoring board (DSMB) will be appointed (see Acknowledgments for details). Plenary DSMB meetings will be held after inclusion of 50, 150, 413 (interim analysis), and 650 patients. The DSMB has access to the unblinded patient data and discusses all serious adverse events. These events will be tabulated and a narrative of the complete case will be provided. All deceased patients will be evaluated by the DSMB for cause of death and whether this is related to a study intervention. After every meeting, the DSMB reports to the trial steering committee. A copy is sent to the ethical committee.

A one-sided interim-analysis of the primary endpoint will be performed when 50% of patients (*n* = 413) have been randomized and discharged. Based on the raw data of every patient, a blinded adjudication committee will determine if the criteria for the primary endpoint are met. The interim-analysis will be performed by a blinded, independent statistician who will report to the DSMB, which has access to unblinded data. The advice of the DSMB will be sent to both the ethics boards and the steering committee. Finally, the steering committee decides whether the FLUYT trial should be continued. The Peto approach is used for beneficial effect. For harm (higher incidence of the primary endpoint in the intervention group) no stopping rule is chosen. The trial will be terminated using an upper stopping boundary at *P* < 0.001.

## Discussion

The FLUYT trial will answer the question of whether combination therapy with periprocedural hydration and rectal NSAIDs significantly lowers PEP incidence compared to NSAID monotherapy. Although several RCTs [12, 30, 31] have investigated the value of periprocedural hydration, they have several shortcomings.

First, only two trials [30, 31] combined periprocedural hydration with standard-of-care PEP prophylactics such as rectal NSAIDs. Because of the solid evidence favoring rectal NSAID use in average- to high-risk patients [4, 7] and an accumulating evidence base for routine use in all patients [32, 33], patients must not be withheld rectal NSAIDs.

Second, the trials had small sample sizes ranging from 26 to 510 patients, increasing the chances of type I and II errors and resulting in a power that is too low to reliably investigate an infrequent complication like PEP. This could explain some unexpected findings that are not in line with the current literature; for instance, two RCTs found no significant difference between placebo and rectal NSAID groups [30, 31]. The FLUYT trial includes 826 patients in two parallel groups, which gives us adequate power to detect a potential difference. Furthermore, our multicenter setting allows for a higher generalizability of results.

Third, many trials deviated from the Cotton criteria to classify PEP [19]. Instead, they defined PEP as abdominal pain and hyperamylasemia, but these symptoms are common after ERCP [34]. For a proper diagnosis according to Cotton, both items should still be present 24 h after ERCP and hospitalization should be prolonged for at least 2 nights. The use of a less stringent PEP definition might result in an overestimation of PEP incidence. In the FLUYT trial, we strictly adhere to the Cotton criteria and all patients are hospitalized for 24 h. Not only does this ensure timely recognition and treatment of adverse events, it also allows for a precise assessment of the primary endpoint by physical examination and measurement of amylase and/or lipase 24 h after ERCP.

The design of the two trials that did use combination therapy do not allow conclusions regarding periprocedural hydration. One trial [30] used a four-arm parallel group design in which all trial arms received 1 L over 30 min prior to ERCP. There was no control group without hydration and, therefore, the additive value of hydration cannot be assessed. The other trial [31] used a conservative hydration schedule of 1 L over 2 h before ERCP and 2 L over 16 h thereafter. This could explain the absence of a significant difference in PEP incidence between the hydration and control groups. The study group design in the FLUYT trial allows a proper evaluation of periprocedural hydration. With respect to fluid type, there is evidence suggesting that lactated Ringer’s solution is preferable in the treatment of acute pancreatitis [18]. Therefore, we chose to compare lactated Ringer’s to a control of normal saline. With respect to fluid volume, the vigorous hydration in our intervention group is expected to result in a significant fluid difference of 1.4 L directly after ERCP and 2.3 L after 9 h (for a 75 kg patient undergoing a 1 h ERCP).

A potential drawback of the FLUYT trial design is the lack of blinding. However, we presume that the large difference in fluid administration will lead to a notable difference in a patient’s urine output. Furthermore, we performed a pilot in which treating staff were blinded for treatment allocation. It was concluded that the blinding procedure would be both unfeasible (with respect to the multicenter setting) and undesirable (with respect to breaking the blinding in case of hydration-related complications). Therefore, a blinded adjudication committee will assess the occurrence of all primary and secondary endpoints.

The 9 h hydration schedule used in most trials raised concerns about its cost-effectiveness. For many hospitals, the schedule could prove difficult to adopt in an outpatient ERCP practice [35]. To address this issue, we will perform a separate cost-effectiveness analysis if our trial finds a significant reduction in PEP incidence in the hydration group.

Several choices in the FLUYT trial design, including hydration schedule, fluid type, and sample size, maximize the power of finding a difference in post-ERCP pancreatitis if such a difference really exists. Therefore, we can answer the question of whether periprocedural hydration provides additional protection against PEP on top of rectal NSAIDs and whether this approach is cost-effective.

## Trial status

The first patient was randomized on June 5, 2015. To date, 515 patients have been randomized and inclusion rate is on schedule. Protocol version 3 is being used and patient recruitment is expected to last until the end of 2019.

## Additional file

Additional file 1: Definitions of secondary endpoints. **Table S1.** Severity of PEP according to Cotton and revised Atlanta criteria. **Table S2.** Local and systemic complications according to (revised) Atlanta criteria. **Table S3.** ERCP-related complications (adopted from Cotton). SPIRIT checklist. (DOCX 40 kb)

## Abbreviations

CRF: Case record form
CCMO: Central committee on research involving human subjects
DSMB: Data safety monitoring board
ERCP: Endoscopic retrograde cholangiopancreatography
ESGE: European Society for Gastrointestinal Endoscopy
FMW: Fictive maximum weight
NSAID: Non-steroidal anti-inflammatory drugs
PD: Pancreatic duct
PEP: Post-ERCP pancreatitis
RCT: Randomized controlled trial
SOP: Standard operating procedure
