# Supplementary material for: Fluid hydration to prevent post-ERCP pancreatitis in average- to high-risk patients receiving prophylactic rectal NSAIDs (FLUYT trial): study protocol for a randomized controlled trial
Source: Trials. 2018 Apr 2;19:207. doi: 10.1186/s13063-018-2583-x (PMC5879873; doi:10.1186/s13063-018-2583-x)
Supplement: Supplementary file 1 — Definitions of secondary endpoints. Table S1. Severity of PEP according to Cotton and revised Atlanta criteria. Table S2. Local and systemic complications according to (revised) Atlanta criteria. Table S3. ERCP-related complications (adopted from Cotton). SPIRIT checklist. (DOCX 40 kb) [file 13063_2018_2583_MOESM1_ESM.docx]

**ADDITIONAL FILES**

**Additional file 1: Definitions of secondary endpoints**

| **Additional table 1: Severity of PEP according to Cotton** [1] **and revised Atlanta criteria** [2] | | | |
| --- | --- | --- | --- |
|  | **Mild** | **Moderate** | **Severe** |
| Cotton criteria | Hospitalization to 2-3 days | Hospitalization to 4-10 days | Hospitalization > 10 days, or hemorrhagic pancreatitis, phlegmon or pseudocyst or intervention (percutaneous drainage or surgery) |
| Revised Atlanta criteria | - No organ failure - No local* or systemic* complications | - Transient organ failure (resolves <48h) and/or - Local or systemic complications without persistent (>48h) organ failure | Persistent (>48h) organ failure   - Single organ failure - Multiple organ failure |

*See table 2 for elaborate definitions

| **Additional table 2: Local and systemic complications according to (revised) Atlanta criteria** [2, 3] | |
| --- | --- |
| **Local complications** [2] | |
| Acute peripancreatic fluid collection | Peripancreatic fluid associated with interstitial oedematous pancreatitis with no associated peripancreatic necrosis. This term applies only to areas of peripancreatic fluid seen within the first 4 weeks after onset of interstitial oedematous pancreatitis and without the features of a pseudocyst. |
| Pancreatic pseudocyst | An encapsulated collection of fluid with a well-defined inflammatory wall usually outside the pancreas with minimal or no necrosis. This entity usually occurs more than 4 weeks after onset of interstitial oedematous pancreatitis to mature. |
| Acute necrotic collection | A collection containing variable amounts of both fluid and necrosis associated with necrotising pancreatitis; the necrosis can involve the pancreatic parenchyma and/or the peripancreatic tissues. |
| Walled-off necrosis | A mature, encapsulated collection of pancreatic and/or peripancreatic necrosis that has developed a well-defined inflammatory wall. WON usually occurs >4 weeks after onset of necrotising pancreatitis. |
| Infected necrosis | The diagnosis of infected necrosis can be suspected by the patient’s clinical course or by the presence of gas within the collection seen on CECT. This extraluminal gas is present in areas of necrosis and may or may not form a gas/fluid level depending on the amount of liquid content present at that stage of the disease. Infected necrosis is also diagnosed by positive culture of necrosis obtained by fine needle aspiration, drainage or necrosectomy. |
| **Systemic complications** [3] | |
| Circulatory organ failure | Systolic blood pressure <90 mmHg, despite adequate fluid resuscitation, or need for inotropic catecholamine support |
| Pulmonary organ failure | PaO_2_ <60 mmHg, despite FiO_2_ of 0.30, or need for mechanical ventilation |
| Renal failure | Creatinine ≥ 177 µmol/l after rehydration or new need for hemofiltration or hemodialysis |
| Gastrointestinal bleeding | >500 ml of blood/24 hours |
| Disseminated intravascular coagulation | Platelet count <100x10^9^/l |
| Severe metabolic disturbance | Calcium < 1.87 mmol/l |

| **Additional table 3: ERCP related complications (adopted from Cotton** [1]**)** | | | |
| --- | --- | --- | --- |
|  | **Mild** | **Moderate** | **Severe** |
| Bleeding | Clinical evidence of bleeding (ie not just endoscopic). Hb-level drop < 1.86mM; no need for transfusion | Transfusion: ≤4 units; no angiographic intervention or surgery | Transfusion: ≥5 units or intervention (angiographic or surgical) |
| Perforation | Possible, or only very slight leak of fluid or contrast dye; treatable by fluids and suction for ≤ 3 days | Any definite perforation treated medically for 4-10 days | Medical treatment for more than 10 days, or intervention (percutaneous or surgical) |
| Infection   \|  \| \| --- \| | - ≥ 38.5°C with chills, without an obvious other cause like a cystitis, pneumonia, thrombophlebitis, etc.), or 39°C without chills, without an obvious cause for fever - *And either:*   - Cholecystodocholithiasis on US, CT, EUS or MRI,   - OR, in the absence of gallstones and/or sludge, a dilated common bile duct on imaging (US, CT or MR) defined as >8mm in patients ≤ 75 years or > 10mm in patients >75 years   - OR progressive cholestasis for at least two consecutive days AND a bilirubin > 2.3 mg/dL (40 μmol/L). | >48h febrile or septic illness requiring > 3 days of hospital treatment or endoscopic or percutaneous intervention | Septic shock or Surgery |

**Additional file 2: SPIRIT checklist**

| Section/item | Item No | Description | Addressed on page number |
| --- | --- | --- | --- |
| **Administrative information** | | |  |
| Title | 1 | Descriptive title identifying the study design, population, interventions, and, if applicable, trial acronym | 1 |
| Trial registration | 2a | Trial identifier and registry name. If not yet registered, name of intended registry | 3, Abstract |
|  | 2b | All items from the World Health Organization Trial Registration Data Set | Throughout entire protocol |
| Protocol version | 3 | Date and version identifier | 15, ‘Trial status’ |
| Funding | 4 | Sources and types of financial, material, and other support | 17, ‘Funding’ |
| Roles and responsibilities | 5a | Names, affiliations, and roles of protocol contributors | 17-23 |
|  | 5b | Name and contact information for the trial sponsor | 18 ‘Coordinating centre, primary sponsor and principal investigators’ |
|  | 5c | Role of study sponsor and funders, if any, in study design; collection, management, analysis, and interpretation of data; writing of the report; and the decision to submit the report for publication, including whether they will have ultimate authority over any of these activities | p. 17 ‘Funding’ |
|  | 5d | Composition, roles, and responsibilities of the coordinating centre, steering committee, endpoint adjudication committee, data management team, and other individuals or groups overseeing the trial, if applicable (see Item 21a for data monitoring committee) | 17-23 |
| Introduction |  |  |  |
| Background and rationale | 6a | Description of research question and justification for undertaking the trial, including summary of relevant studies (published and unpublished) examining benefits and harms for each intervention | 4-5 ‘Background’ |
|  | 6b | Explanation for choice of comparators | 7-8 ‘Treatment arms and co-interventions’ |
| Objectives | 7 | Specific objectives or hypotheses | 4-5 ‘Background’, 8-9 ‘Outcomes’ |
| Trial design | 8 | Description of trial design including type of trial (eg, parallel group, crossover, factorial, single group), allocation ratio, and framework (eg, superiority, equivalence, noninferiority, exploratory) | 6 ‘Study setting’, 9 ‘Randomization’ |
| Methods: Participants, interventions, and outcomes | | |  |
| Study setting | 9 | Description of study settings (eg, community clinic, academic hospital) and list of countries where data will be collected. Reference to where list of study sites can be obtained | 6 ‘Study setting’, 20-21 ‘Participating centers’ |
| Eligibility criteria | 10 | Inclusion and exclusion criteria for participants. If applicable, eligibility criteria for study centres and individuals who will perform the interventions (eg, surgeons, psychotherapists) | 6-7 ‘Eligibility criteria’ |
| Interventions | 11a | Interventions for each group with sufficient detail to allow replication, including how and when they will be administered | 7-8 ‘Treatment arms and co-interventions’ |
|  | 11b | Criteria for discontinuing or modifying allocated interventions for a given trial participant (eg, drug dose change in response to harms, participant request, or improving/worsening disease) | 7-8 ‘Treatment arms and co-interventions’ |
|  | 11c | Strategies to improve adherence to intervention protocols, and any procedures for monitoring adherence (eg, drug tablet return, laboratory tests) | 7-8 ‘Treatment arms and co-interventions’, 10 ‘Data collection methods and follow-up’ |
|  | 11d | Relevant concomitant care and interventions that are permitted or prohibited during the trial | 7-8 ‘Treatment arms and co-interventions’ |
| Outcomes | 12 | Primary, secondary, and other outcomes, including the specific measurement variable (eg, systolic blood pressure), analysis metric (eg, change from baseline, final value, time to event), method of aggregation (eg, median, proportion), and time point for each outcome. Explanation of the clinical relevance of chosen efficacy and harm outcomes is strongly recommended | 8-9 ‘Outcomes’, 11 ‘Primary analysis’, ‘Supplement 1’ |
| Participant timeline | 13 | Time schedule of enrolment, interventions (including any run-ins and washouts), assessments, and visits for participants. A schematic diagram is highly recommended (see Figure) | Figure 1 |
| Sample size | 14 | Estimated number of participants needed to achieve study objectives and how it was determined, including clinical and statistical assumptions supporting any sample size calculations | 9 ‘Sample size calculation’ |
| Recruitment | 15 | Strategies for achieving adequate participant enrolment to reach target sample size | 8 |
| **Methods: Assignment of interventions (for controlled trials)** | | |  |
| Allocation: |  |  |  |
| Sequence generation | 16a | Method of generating the allocation sequence (eg, computer-generated random numbers), and list of any factors for stratification. To reduce predictability of a random sequence, details of any planned restriction (eg, blocking) should be provided in a separate document that is unavailable to those who enrol participants or assign interventions | 11 ‘Randomization’ |
| Allocation concealment mechanism | 16b | Mechanism of implementing the allocation sequence (eg, central telephone; sequentially numbered, opaque, sealed envelopes), describing any steps to conceal the sequence until interventions are assigned | 9 ‘Randomization’ |
| Implementation | 16c | Who will generate the allocation sequence, who will enrol participants, and who will assign participants to interventions | 9 ‘Randomization’ |
| Blinding (masking) | 17a | Who will be blinded after assignment to interventions (eg, trial participants, care providers, outcome assessors, data analysts), and how | 10 ‘Blinding’, 13 ‘Discussion’ |
|  | 17b | If blinded, circumstances under which unblinding is permissible, and procedure for revealing a participant’s allocated intervention during the trial | Not applicable: unblinded |
| **Methods: Data collection, management, and analysis** | | |  |
| Data collection methods | 18a | Plans for assessment and collection of outcome, baseline, and other trial data, including any related processes to promote data quality (eg, duplicate measurements, training of assessors) and a description of study instruments (eg, questionnaires, laboratory tests) along with their reliability and validity, if known. Reference to where data collection forms can be found, if not in the protocol | 9 ‘Data collection methods’ |
|  | 18b | Plans to promote participant retention and complete follow-up, including list of any outcome data to be collected for participants who discontinue or deviate from intervention protocols | 9 ‘Data collection methods’ |
| Data management | 19 | Plans for data entry, coding, security, and storage, including any related processes to promote data quality (eg, double data entry; range checks for data values). Reference to where details of data management procedures can be found, if not in the protocol | 10 ‘Data collection methods’ |
| Statistical methods | 20a | Statistical methods for analysing primary and secondary outcomes. Reference to where other details of the statistical analysis plan can be found, if not in the protocol | 11-12 ‘Statistical methods’ |
|  | 20b | Methods for any additional analyses (eg, subgroup and adjusted analyses) | 11-12 ‘Statistical methods’ |
|  | 20c | Definition of analysis population relating to protocol non-adherence (eg, as randomised analysis), and any statistical methods to handle missing data (eg, multiple imputation) | 11-12 ‘Statistical methods’ |
| **Methods: Monitoring** | | |  |
| Data monitoring | 21a | Composition of data monitoring committee (DMC); summary of its role and reporting structure; statement of whether it is independent from the sponsor and competing interests; and reference to where further details about its charter can be found, if not in the protocol. Alternatively, an explanation of why a DMC is not needed | 12 ‘Safety’, 20 ‘DSMB’ |
|  | 21b | Description of any interim analyses and stopping guidelines, including who will have access to these interim results and make the final decision to terminate the trial | 12 ‘Safety’ |
| Harms | 22 | Plans for collecting, assessing, reporting, and managing solicited and spontaneously reported adverse events and other unintended effects of trial interventions or trial conduct | 12 ‘Safety’ |
| Auditing | 23 | Frequency and procedures for auditing trial conduct, if any, and whether the process will be independent from investigators and the sponsor | Not applicable |
| Ethics and dissemination | | |  |
| Research ethics approval | 24 | Plans for seeking research ethics committee/institutional review board (REC/IRB) approval | 17 ‘Ethical Approval…’ |
| Protocol amendments | 25 | Plans for communicating important protocol modifications (eg, changes to eligibility criteria, outcomes, analyses) to relevant parties (eg, investigators, REC/IRBs, trial participants, trial registries, journals, regulators) | 17 ‘Ethical Approval…’: standard according to Dutch Medical Research Involving Human Subjects Act (WMO) |
| Consent or assent | 26a | Who will obtain informed consent or assent from potential trial participants or authorised surrogates, and how (see Item 32) | 17 ‘Ethical Approval…’: standard according to Dutch Medical Research Involving Human Subjects Act (WMO) |
|  | 26b | Additional consent provisions for collection and use of participant data and biological specimens in ancillary studies, if applicable | Not applicable |
| Confidentiality | 27 | How personal information about potential and enrolled participants will be collected, shared, and maintained in order to protect confidentiality before, during, and after the trial | 17 ‘Ethical Approval…’: standard according to Dutch Medical Research Involving Human Subjects Act (WMO) |
| Declaration of interests | 28 | Financial and other competing interests for principal investigators for the overall trial and each study site | 17 ‘Competing interests’ |
| Access to data | 29 | Statement of who will have access to the final trial dataset, and disclosure of contractual agreements that limit such access for investigators | 17 ‘Availability of data…’ |
| Ancillary and post-trial care | 30 | Provisions, if any, for ancillary and post-trial care, and for compensation to those who suffer harm from trial participation | 17 ‘Ethical Approval…’: standard according to Dutch Medical Research Involving Human Subjects Act (WMO) |
| Dissemination policy | 31a | Plans for investigators and sponsor to communicate trial results to participants, healthcare professionals, the public, and other relevant groups (eg, via publication, reporting in results databases, or other data sharing arrangements), including any publication restrictions | No publication restrictions |
|  | 31b | Authorship eligibility guidelines and any intended use of professional writers |  |
|  | 31c | Plans, if any, for granting public access to the full protocol, participant-level dataset, and statistical code | 17 ‘Availability of data…’ |
| Appendices |  |  |  |
| Informed consent materials | 32 | Model consent form and other related documentation given to participants and authorised surrogates |  |
| Biological specimens | 33 | Plans for collection, laboratory evaluation, and storage of biological specimens for genetic or molecular analysis in the current trial and for future use in ancillary studies, if applicable | Not applicable |

**REFERENCE LIST**

1. Cotton PB, Lehman G, Vennes J, Geenen JE, Russell RC, Meyers WC, et al. Endoscopic sphincterotomy complications and their management: an attempt at consensus. Gastrointest Endosc. 1991;37:383–93.

2. Banks PA, Bollen TL, Dervenis C, Gooszen HG, Johnson CD, Sarr MG, et al. Classification of acute pancreatitis--2012: revision of the Atlanta classification and definitions by international consensus. Gut. 2012;:102–11.

3. Bradley EL. A clinically based classification system for acute pancreatitis. Summary of the International Symposium on Acute Pancreatitis, Atlanta, Ga, September 11 through 13, 1992. Arch Surg. 1993;128:586–90.
